# Non-coding RNA-Associated Therapeutic Strategies in Atherosclerosis

**DOI:** 10.3389/fcvm.2022.889743

**Published:** 2022-04-25

**Authors:** Yuyan Tang, Huaping Li, Chen Chen

**Affiliations:** ^1^Division of Cardiology, Tongji Hospital, Tongji Medical College, Huazhong University of Science and Technology, Wuhan, China; ^2^Hubei Key Laboratory of Genetics and Molecular Mechanisms of Cardiological Disorders, Wuhan, China

**Keywords:** non-coding RNA, atherosclerosis, inflammatory, biomarker, therapy

## Abstract

Atherosclerosis has been the main cause of disability and mortality in the world, resulting in a heavy medical burden for all countries. It is widely known to be a kind of chronic inflammatory disease in the blood walls, of which the key pathogenesis is the accumulation of immunologic cells in the lesion, foam cells formation, and eventually plaque rupture causing ischemia of various organs. Non-coding RNAs (ncRNAs) play a vital role in regulating the physiologic and pathophysiologic processes in cells. More and more studies have revealed that ncRNAs also participated in the development of atherosclerosis and regulated cellular phenotypes such as endothelial dysfunction, leukocyte recruitment, foam cells formation, and vascular smooth muscle cells phenotype-switching and apoptosis. Given the broad functions of ncRNAs in atherogenesis, they have become potential therapeutic targets. Apart from that, ncRNAs have become powerful blueprints to design new drugs. For example, RNA interference drugs were inspired by small interfering RNAs that exist in normal cellular physiologic processes and behave as negative regulators of specific proteins. For instance, inclisiran is a kind of RNAi drug targeting PCKS9 mRNA, which can lower the level of LDL-C and treat atherosclerosis. We introduce some recent research progresses on ncRNAs related to atherosclerotic pathophysiologic process and the current clinical trials of RNA drugs pointed at atherosclerosis.

## Introduction

Cardiovascular diseases (CVDs) have been the main cause of death worldwide for decades, and atherosclerosis is a common etiology for various CVDs, such as myocardial infarctions and strokes (1). Therefore, it is of great value to figure out the pathophysiologic progression and the treatments for atherosclerosis. It is widely acknowledged that atherosclerosis is a chronic inflammatory disease characterized by the accumulation of plenty of immune cells, typically monocyte-derived macrophages, in the subendothelial space of arterial walls composed of three layers, namely intima, media, and adventitia (2).

The pre-atherosclerotic lesion in humans is thought to be diffuse intima thickening at the atherosclerosis-prone areas of arterials caused by disturbed blood flow, occurring even at an early age (3). The vascular smooth muscle cells from the media migrate into the intima and produce a negatively charged extracellular matrix that can interact with positively charged apoB-containing lipoproteins (4). The lipid retention in the subendothelial space initiates endothelial dysfunction and endothelial activation, resulting in a higher permeability of the endothelium and the production of inflammatory cytokines and adhesion molecules (5). Besides, the lipid stuck in the intima is susceptible to be modulated especially oxidation, resulting in oxidized lipid (6). Therefore, the monocytes in circulation are recruited to the lesion and enter the intima, where they differentiate into macrophages, take lipids, become foam cells, and are trapped here (2). The vascular smooth muscle cells continue to migrate into the intima and proliferate as well as synthesize collagen, which promotes the formation of fibrous cap (7). However, in advanced plaques, apoptosis and necrosis occur more frequently while proliferation and efferocytosis are suppressed, making the plaque an acellular lesion and easy to rupture.

The key cells in atherogenesis are endothelial cells, macrophages, and vascular smooth muscle cells. The vascular endothelium, composed of a single layer of endothelial cells (EC) and coating the inner surface of blood vessels, plays a vital role in the initiation of atherosclerosis. Studies have illustrated that high-risk factors for atherosclerosis lead to endothelial cell dysfunction and proinflammation activation eventually promoting atherogenesis. For example, the low shear stress at lesion-prone regions, arterial branches, and curvatures reduces eNOS expression and can also induce ROS by suppressing AT1R/eNOS/NO pathway (8, 9), both of which can impair endothelial permeability. The co-pathway of endothelial cell dysfunction is the activation of NF-κB (5, 10). It stimulates the synthesis of chemokines such as IL-8 and MCP-1 to trigger leukocyte recruitment (11). Furthermore, It can induce the expression of adhesion molecules on the surface of endothelial cells (12) such as VCAM-1 (13), ICAM1, and E-selectin (11) which initiate leukocyte adhesion. The key mechanism of atherosclerosis is the formation of foam cells in plaques as a result of macrophages' uptake of ox-LDL. The formation of foam cells is associated with impaired lipid metabolism and accumulation of lipid within macrophages. Scavenger receptor class A (SR-A), cluster of differentiation 36 (CD36), and lectin-like oxidized low-density lipoprotein receptor-1 (LOX-1) are upregulated in the atheroma (14, 15), mediating the uptake of oxLDL, promoting lipid accumulation within the macrophages, and finally exacerbating atherosclerosis (16, 17). In addition to increased intake of lipid, cholesterol efflux is also impaired in atherosclerosis contributed by lipid accumulation. The transporters mediating cholesterol efflux, such as ABCA1 and ABCG1, are decreased in the macrophage-derived foam cells (18). Consistently, deficiency of ABCA1 or ABCG1 contributes to damaged cholesterol efflux (19, 20), while their upregulation protects macrophages from forming foam cells and slows down plaques' enlargement (21, 22). The vascular smooth muscle cells (VSMCs) are normally located in the medial layer of blood vessels, whose functions are controlling vessels tone by contraction and producing extracellular matrix (ECM) (23). With the development of atherogenesis, VSMCs switch their phenotype from contractile to synthetic, partially because of oxLDL stimulation (24). At the early stage of atherosclerosis, VSMCs migrate from media and proliferate in the intima, but cell cycle and growth are arrested reversely at advanced plaques, which is termed as VSMC senescence (25, 26). Apart from decreased proliferation rate, VSMCs turn to senescence-associated secretory phenotype (SASP), characterized by secreting several pro-inflammatory mediators such as IL-6, IL-8, and producing metalloprotease, ending up with vulnerable atheroma and plaque rupture (27, 28). VSMCs apoptosis and necrosis are rare at the beginning but become common at advanced plaques (29, 30). Loss of VSMCs and their ability to synthesize ECM leads to plaque vulnerability (31).

Non-coding RNAs are RNAs that lack the ability to encode peptides or proteins, some of which function as regulators of other genes' expression. They have been potential therapeutic targets in the past few years because they can regulate protein production (32). Since ncRNAs play an important role in many diseases such as cancers, neurological diseases, and cardiovascular diseases involving atherosclerosis (33), pre-clinical studies and clinical trials for drugs targeting ncRNAs involved in atherogenesis are emerging currently (34).

## Non-Coding RNAs in Atherosclerosis

With the development of full genome sequencing technology, it is found that although the major part of the human genome is transcriptionally active, only a little proportion of it can encode protein and the remaining comprises non-coding RNAs (ncRNAs) (35). Several ncRNAs are currently well studied, like microRNAs (miRNAs), long non-coding RNAs (lncRNAs), and circular RNA (circRNAs; Table 1). miRNAs are at the size of ~22 nucleotides and regulate gene expression post-transcriptionally. In mammalian cells, they usually are incorporated into the RNA-induced silencing complex (RISC) and they guide the complex to the mRNA in which the 3′ UTR region is complementary to them, resulting in mRNA cleavage or translation regression (82). When it comes to lncRNAs, they are characterized as transcripts longer than 200 nt and without the ability to encode proteins. After being transcribed from either intergenic regions or protein-coding regions, both in the sense or antisense direction, they function in different ways. For example, they can act as a scaffold to help the assembly of protein complexes (83). They can also serve as a decoy for RNA-binding proteins or miRNAs such as transcription factors and prevent their functions. The ability to bind miRNA is called the “sponge effect”. Besides, after binding target proteins, they can guide the complex to specific locations, which is called “guides” (84). CircRNAs are mostly generated from precursor mRNAs undergoing back splicing reactions where a downstream splice donor (5′ splice sites) is joined to a splice acceptor (3′ splice site) of the intron because of its slow slicing. Once generated, they are resistant to exonucleases cleavage and become stable in cells. The functions of circRNAs remain in research, but some of them can also serve as decoys or sponges for miRNAs. As a result, the number of free targeting miRNAs decreases, weakening their blocking functions (85).

**Table 1:** Non-coding RNAs in atherosclerosis.

**ncRNA name**	**Model**	**Functions**	**Pro-/anti-atherogenesis**	**Reference**
hsa_circ_0003575	HUVECs	Suppressing ECs proliferation and promoting apoptosis	Not accessed	(36)
NEXN-AS1	ECs	Reducing endothelial inflammatory activation	Anti-atherogenesis	(37)
SENCR	HUVECs and HCAECs	Stabilizing membrane integrity and permeability of ECs	Anti-atherogenesis	(38, 39)
LncRNA-FA2H-2-MLKL	ECs and VSMCs	Inhibiting autophagy and inflammation	Anti-atherogenesis	(40, 41)
miR-1a-3p and miR-1b-5p	ECs	Activating endothelial inflammation	Pro-atherogenesis	(42)
miR-181a-5p and miR-181a-3p	ECs	Inhibiting endothelial inflammation	Anti-atherogenesis	(43)
miR-126-5p	ECs	Suppressing endothelial inflammatory activation and promoting ECs proliferation	Anti-atherogenesis	(44, 45)
circANRIL	VSMCs and macrophages	Preventing ribosome biogenesis by inhibiting proliferation	Anti-atherogenesis	(46–48)
circ_Lrp6	VSMCs	Inhibiting migration, proliferation and phenotype-switching	Anti-atherogenesis	(49)
circACTA2	VSMCs	Regulating SMCs contraction	Not accessed	(50)
SMILR	VSMCs	Promoting proliferation and mitosis	Pro-atherogenesis	(51, 52)
CARMN	VSMCs	Inhibiting VSMCs phenotype switching	Anti-atherogenesis	(53, 54)
miR-22	VSMCs	Suppressing VSMCs phenotyping switching	Not accessed	(55, 56)
miR-128	VSMCs	Suppress VSMCs proliferation, migration and phenotype switching	Anti-atherogenesis	(57)
miR-145	VSMC	Preventing VSMCs phenotype-switching	Anti-atherogenesis	(58–61)
MALAT1	Macrophages	Preventing inflammatory factors production and leukocytes retention	Anti-atherogenesis	(62–64)
MeXis	Macrophages	Promoting cholesterol efflux	Anti-atherogenesis	(65, 66)
MAARS	Macrophages	Inducing apoptosis and impairing efferocytosis	Pro-atherogenesis	(67, 68)
miR-181b	Macrophages	Stabilizing fibrous cap	Pro-atherogenesis	(69, 70)
miR-34a	Macrophages	Impairing cholesterol efflux	Pro-atherogenesis	(71–73)
miR-30c-5p	Macrophages	Promoting apoptosis	Anti-atherogenesis	(74)
miR-33	Macrophages and hepatocytes	Impairing cholesterol efflux	Pro-atherogenesis	(75–78)
miR-144	Macrophages and hepatocytes	Impairing cholesterol efflux	Not accessed	(79, 80)
CHROME	Macrophages and hepatocytes	Impairing cholesterol efflux	Not accessed	(81)

## Non-Coding RNAs in Endothelial Cells

### miR-1a-3p and miR-1b-5p

Non-alcoholic fatty liver disease (NAFLD), associated with increased triglycerides and decreased HDL in the blood, was identified to be related to subclinical atherosclerosis, regardless of traditional risk factors and metabolic syndrome (42). To clarify the relationship between the two diseases, the exosomes secreted from steatotic hepatocytes were analyzed by miRNA deep sequencing, among which the clusters of miR-1, miR-1a-3p, and miR-1b-5p were the most significantly raised miRNAs. ECs were confirmed to absorb miR-1 involved in exosomes from hepatocytes by co-culture. Subsequent experiments showed that miR-1 mimics were able to activate endothelial inflammation using the enhanced expression of E-selectin, ICAM-1, and VCAM-1 at both mRNA and protein levels. *In vivo* studies revealed that miR-1 knockdown by antagomiR-1 regressed endothelial inflammatory activation as well as the lesion areas, revealing a new therapeutic target for atherosclerosis (86).

### miR-181a-5p and miR-181a-3p

Both miR-181a-5p and miR-181a-3p were found to descend in atherosclerotic lesions of ApoE^−/−^ mice fed a high-fat diet as well as the plasma of patients with coronary arterial diseases, suggesting the potential role of the two miRNAs in atherogenesis. The overexpression of miR-181a-5p and miR-181a-3p in ApoE^−/−^ mice attenuated the plaque size as expected. Further studies showed that the gain-of-function experiment suppressed inflammatory genes, such as ICAM-1 and VCAM-1, as well as leukocytes infiltration in aortic intima rather than impacting lipid metabolism. To explore the molecular mechanism, algorithms were used to predict their potential targets, among which NEMO for miR-181a-3p and TAB2 for miR-181a-5p, involved in the NF-κB signaling pathway in ECs, were confirmed by luciferase reporter assay. As a result, miR-181a-5p and miR-181a-3p were decreased in atherogenesis to activate endothelial inflammation (43).

### miR-126-5p

Previous studies exhibited that the pro-atherogenic triglyceride-rich lipoproteins from subjects having a high-fat meal suppressed miR-126 expression in ECs and promoted VCAM-1 expression as well as monocyte adhesion, suggesting the potential anti-atherosclerotic role of miR-126 (44). Another research showed that apart from suppression and endothelial inflammatory activation, miR-126-5p, rather than miR-126-3p, could promote ECs' proliferation by decreasing Notch1 inhibitor delta-like 1 homolog (Dlk1) expression so as to prevent plaque formation, which was identified in Mir126^−/−^ApoE^−/−^ mice. However, disturbed laminar flow downregulated miR-126 at atherosclerosis-prone areas, abolishing its protective effect (45).

### hsa_circ_0003575

To identify circRNAs participating in atherosclerosis, a circRNA microarray analysis was performed in HUVECs stimulated by oxLDL, among which hsa_circ_0003575 was mostly upregulated. *In vitro*, loss-of-function experiments showed that hsa_circ_0003575 was able to suppress ECs' proliferation and promote apoptosis. The bioinformatic assays revealed that hsa_circ_0003575 might serve as a miRNAs sponge for miR-199-3p, miR-9-5p, miR-377-3p, and miR-141-3p. However, more *in vivo* evidence is still required for the precise mechanism of hsa_circ_0003575 in atherosclerosis (36).

### NEXN-AS1

A microarray analysis was performed to identify dysregulated genes in atherosclerosis lesions, among which the NEXN gene, as well as the lncRNA gene, NEXN-AS1 was significantly decreased. *In vivo* experiment revealed that NEXN-AS1 lncRNA could enhance NEXN expression in ECs, and the overexpression of NEXN-AS1 reduced endothelial inflammatory activation by inhibiting the NF-κB pathway. Nevertheless, the protective functions of NEXN-AS1 disappeared when NEXN was knocked down. The anti-atherogenic role of NEXN was determined with the NEXN^+/−^ ApoE^−/−^ mice. After a Western diet for 12 weeks, the NEXN^+/−^ ApoE^−/−^ mice presented a heavier plaque burden and a stronger tendency to rupture than the controls. As a result, NEXN-AS1 plays a protective role in atherosclerosis and has the potential to be a treatment target (37).

### SENCR

SENCR was found to be rich in the human coronary artery smooth muscle cells (HCASMC) by RNA-sequencing. Further studies showed that SENCR knockdown downregulated the contractile gene expression in HCASMCs and promoted proliferation and migration, on the contrary, revealing its influence on phenotype switching of VSMCs (38). Another research explored its impact on endothelial cells as the lncRNA was also expressed in them (38). Exposed to laminar shear stress, a protective factor for atherosclerosis, SENCR level was elevated in both human umbilical vein endothelial cells (HUVEC) and human coronary artery endothelial cells (HCAEC), but not reversely decreased when subjected to disturbed shear stress. The knockdown studies showed that the loss of SENCR impaired membrane integrity and the permeability of endothelial cells. Further experiments exhibited that SENCR was directly bound to cytoskeletal-associated protein 4 (CKAP4) and stabilized adherens junctions in ECs (39). Despite the lack of direct evidence, the results above illustrate there is a high possibility that SENCR is associated with atherogenesis.

### lncRNA-FA2H-2-MLKL

It is broadly acknowledged that oxLDL is one of the strongest inflammatory triggers for atherosclerosis, and autophagy is the protective pathway of cells faced with stress to survive. Studies revealed that oxLDL decreased the activity of enzyme mature-Cathepsin D, as well as its number, accounting for decreased lysosome activity, which is partially contributed to impaired autophagic flux and less cell survival in atherogenesis. To figure out the molecular mechanism, a microarray analysis was carried out to find out dysregulated genes, among which MLKL, responsible for autophagy and inflammation (40), was overexpressed in ECs and SMCs. Subsequent analysis showed that lncRNA-FA2H-2, which was decreased in human advanced atherosclerotic plaque compared with normal arterial intima, was adjacent to MLKL. Dual-luciferase assays exhibited that lncRNA-FA2H-2 could directly bind to the MLKL promotor and downregulate it. Further lncRNA-FA2H-2 knockdown experiments in ApoE-deficient mice showed that the size of plaques was enlarged and the cell death was enhanced, followed by an increased level of MLKL protein, suggesting the protective effect of lncRNA-FA2H-2 in atherosclerosis (41).

## Non-Coding RNAs in Vascular Smooth Muscle Cells

### miR-22

Previous studies showed that miR-22 was identified to be upregulated in embryonic stem cells and adventitia progenitor cells, responsible for SMC differentiation through regressing MECP2 expression (55). The miRNA was found to be significantly decreased in atherosclerotic plaques, suggesting that it might function in atherogenesis by manipulating VSMC plasticity. Overexpression and silencing experiments revealed that miR-22 was able to suppress VSMC proliferation and migration as well as promote VSMC specific markers expression such as SMαA, SM22α, SM-myh11, and SMTN-B, suggesting its function for maintaining VSMC contractile phenotype. To access its therapeutic potential, miR-22 AgomiR and LNA-miR-22 were immediately transfected into mice with injured femoral arteries caused by the wire. The results exhibited that miR-22 could prevent arterials from neointima hyperplasia by suppressing VSMC phenotyping switching (56). However, its direct protective effect on atherosclerosis remains to be explored.

### miR-128

miR-128 was identified as a conversed miRNA modulated by VSMC DNA methylation status by miRNA profiling since it was highly expressed, strongly changed, and less investigated in VSMC biology. Overexpression experiments revealed that miR-128 was capable of suppressing VSMC proliferation, migration, as well as keeping VSMCs in contractile phenotype, suggesting its protective effect for VSMC de-differentiation. To explore the molecular mechanism, two different kinds of bioinformatic algorithms were taken advantage of to look for the targets of miR-128, with KLF4 identified by luciferase reporter assays. The therapeutic potential for it was subsequently investigated in ApoE^−/−^ mice with a perivascular carotid collar to create stenosis. The results showed that miR-128 overexpression inhibited VSMC proliferation and neointima formation, identifying its protective role (57).

### miR-145

miR-145 were identified to be expressed specifically in the heart and smooth muscle with the use of transgenic mice and Bluo-gal staining. *In vitro* experiments exhibited that miR-145 could determine the fate of the VSMC and keep them in a contractile phenotype rather than a proliferative and synthetic phenotype (58). Clinical studies revealed that miR-145 was significantly reduced in patients with coronary arterial diseases (59). Given its protective effects on the VSMC, several therapeutic strategies focused on raising the level of miR-145 in patients. In the beginning, the prevention effects of miR-145 were explored. ApoE^−/−^ mice were injected with miR-145 lentivirus regulated by the SMC-specific promoter SM22α, followed by 12 weeks high-fat diet. As expected, SMC-specific miR-145 protected arteries from atherogenesis by reducing the size of the lesion, stabilizing the plaque, and hindering macrophage infiltration (60). Another study utilized a novel nanoparticles technology to ensure that miR-145 was precisely transfected into VSMCs, namely the miR-145 micelles. To figure out its therapeutic effect, both an early-stage and an advanced-stage atherosclerotic ApoE^−/−^ mice were treated with the miR-145 micelles, resulting in the inhibition of phenotype switching of VSMCs and preventing atherosclerotic progression (61).

### circANRIL

Previous research showed that linear ANRIL transcripts at chromosome 9p21 were strongly associated with chromosome 9p21 (46). As the gene locus at chromosome 9p21 was also transcribed as circANRIL, which was more highly expressed in cells than linANRIL RNA and also more stable, circANRIL was worthy of investigation in atherosclerosis. *In vitro* overexpression and knockdown experiments exhibited that circANRIL could promote apoptosis and suppress proliferation in HEK-293 cells, SMCs, and macrophages. Neither did circANRIL serve as a miRNA sponge nor was it bound to Argonaute 2 to degrade mRNAs mediated by miRNAs. It is directly bound to pescadillo homolog 1, which is responsible for 60S preribosomal assembly, preventing ribosome biogenesis. Further studies in human atherosclerotic plaques confirmed that high circANRIL levels were strongly correlated with the accumulation of 32S and 36S pre-rRNA (47). A recent study also showed that circANRIL was significantly negatively correlated with CAD risk and severity, suggesting its value for diagnosis and prognosis (48).

### circ_Lrp6

It is widely known that circular RNAs can act as miRNAs sponges, which can downregulate miRNA expression, impacting cell biology as well. By RNA sequencing and bioinformatic analysis, circ_Lrp6 in VSMCs was identified because of the abundant binding sites for miR-145 and its conservation between humans and mice. With methods of RNA immunoprecipitation and competitive luciferase assays, circ_Lrp6 was confirmed to be a miRNA sponge for miR-145. *In vitro* silencing and overexpression experiments exhibited that circ_Lrp6 reversed miR-145-mediated inhibition of migration, proliferation, and VSMC phenotype switching. *In vivo* study further identified the pro-proliferative effect of circ_Lrp6. By injecting ApoE^−/−^ with a perivascular carotid collar with circ_Lrp6-shRNA, the neointima hyperplasia was significantly attenuated (49).

### circACTA2

In VSMCs, circACTA2 expression was induced by NRG-1-ICD, the intracellular domain of Neuregulins, that was able to bind to α-SMA and promote the cytoskeletal organization as a result. By luciferase assay and pulldown assay, circACTA2 was identified to bind to miR-548f-5p and reduce its expression. Subsequent experiments revealed that miR-548f-5p could bind to 3′-UTR of α-SMA and inhibit its expression. Therefore, the NRG-1-ICD/circACTA2/miR-548f-5p axis was built to regulate SMC contraction. Further *in vivo* studies exhibited that circACTA2 and its interaction with miR-548f-5p were decreased, while miR-548f-5p was increased in intimal hyperplastic arteries, suggesting the potential effect of circACTA2 in atherosclerosis (50).

### SMILR

After being treated with IL1α and platelet-derived growth factor (PDGF) for 72 h to induce proliferation and inflammation in human saphenous vein-derived smooth muscle cells, RNA-seq was performed to identify subsequently varying lncRNAs. Among them, SMILR was the most differently expressed one with a particular location in the smooth muscle cells. *In vitro* experiments showed that SMILR was related to SMC proliferation and increased in human atherosclerotic lesions vs. neighboring artery tissues (51). Further molecular mechanisms were identified by fluorescence ubiquitin cell cycle indicator viral system and SMILR pulldowns, revealing that SMILR was able to promote mitosis by regulating late mitotic protein CENPF mRNA. Since SMILR was a human-specific lncRNA, the usage of animal models was limited. To access the therapeutic potential, a siRNA approach was used in vein grafts before grafting, resulting in reduced proliferation of SMCs (52).

### CARMN

CARMN was identified as a strongly expressed SMC-specific lncRNA by transcriptome analysis and was highly conserved in mice as well, which was confirmed in the GFP KI Reporter Mouse Model that made the location of CARMN in cells visible. Analysis of scRNA-seq data from human atherosclerotic arteries showed that CARMN-positive cells, as well as CARMN levels, descended, suggesting its role in atherogenesis (53). The molecular mechanism was explored in another study. *In vivo* CARMN knockdown experiments combined with RNA sequencing analysis revealed that the dysregulated genes were strongly associated with VSMC phenotype switching, thereby promoting atherosclerosis. As CARMN was located exactly upstream of miR-143 and−145, experiments were carried out to figure out whether the effect of CARMN was dependent on the two miRNAs. On silencing CARMN by GapmeR strategy and overexpressing miR-143 and−145 by mimics at the same time, hCASMCs migration and VSMC identifying markers were observed to restore, whereas hCASMCs proliferation remained increased, revealing the independent role of the lncRNA in regulating VSMC proliferation. Further *in vivo* studies showed CARMN was a protective non-coding RNA in atherogenesis consistently (54).

## Non-Coding RNAs in Macrophages

### miR-181b

miR-181b was found to rise in unstable atherosclerotic plaques compared with stable ones, which was associated with an obvious fall in TIMP-3 protein. TIMP-3 was an inhibitor of matrix metalloproteinases (MMPs), a variety of proteases produced by macrophages. A previous study showed that in the fibrous cap of human atheroma, TIMP-3 was increased to prevent ECM from proteolysis by inhibiting MMPs (69). Thus, miR-181b may serve as a pro-atherogenic miRNA. To confirm the hypothesis, ApoE-deficient mice burdened with atherosclerotic lesions were treated with miR-181b inhibitor, and the TIMP-3 levels, proteolytic activity, and the lesion area were tested. As expected, macrophage TIMP-3 expression was enhanced after the inhibition of miR-181b, followed by attenuated lesion progression. However, this kind of protective effect was abolished when Timp3 was knocked out at the same time, revealing that miR-181b deteriorated atherosclerosis by suppressing TIMP-3 expression (70).

### miR-34a

miR-34a is widely expressed in macrophages, endothelial cells, smooth muscle cells (SMCs), and hepatocytes. The previous study showed that miR-34a was highly induced in hepatocytes in NAFLD, which suppressed hepatic HNF4α in both mRNA and protein levels, resulting in a fatty liver by preventing VLDL from secreting but attenuating atherosclerotic plaques in both ApoE^−/−^ and Ldlr^−/−^ mice on the contrary (71). In addition, miR-34a was also induced in human and mice's atheroma, so its influence on atherogenesis was further explored later. In VSMCs, miR-34a was able to promote cell senescence and vascular calcification by decreasing Axl and SIRT1 (72). In macrophages, miR-34a was targeted to ABCA1 and ABCG1, which resulted in impaired cholesterol efflux and macrophage-specific miR-34a deletion, which could prevent atherogenesis. Given the contradictory effect on different tissues, a global miR-34a deficient model was introduced to access its impact in total. Dyslipidemia, atherosclerosis, and NAFLD were all reserved subsequently, suggesting that miR-34a mainly played a negative role in atherogenesis (73).

### miR-30c-5p

To identify whether miRNAs were able to predict and promote atherosclerotic progression, 99 volunteers from the PLIC study free from atherosclerosis were randomly selected to measure their plasma miRNA levels at first, among whom 44 participants developed atherosclerosis after a 5-year follow-up and another 29 did after 6.5 years. Comparing the miRNA basal line at the beginning, a total of 7 miRNAs were picked, among which miR-30c-5p had the best ability to discriminate patients with plaque burden with the highest AUC and to predict atherosclerotic progression. Further, *in vitro* experiments revealed that plasma miR-30c-5p contained in microparticles secreted from macrophages was significantly reduced when treated with oxLDL, resulting from CD36 activation and inhibition of Dicer. Consequently, miR-30c-5p in cells fell, resulting in the upregulation of its target Caspase-3 and inducing IL-6β releasing, as well as the apoptosis of macrophages. *In vivo* study also showed that the lack of miR-30c-5p worsened endothelial healing response to injury, regarded as the first step of atherogenesis (74).

### MALAT1

The previous study identified the association between the long non-coding RNA MALAT1 and cardiovascular innate immunity, showing its potential regulating effect on monocytes and macrophages (62). Two pieces of research revealed the protective function of MALAT1 in macrophages at the same time. One of the two utilized heterozygous MALAT1-deficient ApoE^−/−^ mice to access the influence of the lncRNA on atherosclerosis. Even with a normal diet, the knockdown mice showed a progressive lesion and an increased level of inflammatory factors such as IFN-γ, TNF, and IL6, revealing that the function of MALAT1 is related to inflammation (63). The other study also measured the plaque burden of ApoE^−/−^ Malat1^−/−^ mice fed with a high-fat diet for 12 weeks. However, the lesion showed no significant difference compared with controls except for higher CD45^+^ leukocytes retention, differing from the former study, partially because of compensatory upregulation of the adjacent lncRNA NEAT1. To identify the impact of MALAT1 on inflammatory cells, the bone marrow of ApoE^−/−^ Malat1^−/−^ was transplanted into ApoE^−/−^ Malat1^+/+^ mice, resulting in enlarged atherosclerotic plaques where more macrophage infiltrated. Further experiments showed that MALAT1 served as a microRNA sponge for miR-503 related to enhanced adhesion ability to activate endothelial cells. Moreover, MALAT1 is highly decreased in human atheroma, further demonstrating its anti-atherogenic function (64).

### MeXis

The liver X receptors (LXRs) are transcriptional factors that promote the expression of genes related to reverse cholesterol transport containing Abca1 when intracellular cholesterol was elevated (65). It was noted that ABCA1 responsible for cholesterol efflux was particularly increased in macrophages in comparison with other cell types when treated with synthetic LXR agonist. By transcriptional profiling, several kinds of lncRNAs were also found to be increased, among which the lncRNA MeXis was most raised and of which the promoter was able to bind with LXRs, suggesting that LXRs could regulate cholesterol efflux in macrophage by controlling the transcription of MeXis. *In vitro* experiments exhibited that LXRs did induce the expression of ABCA1 by promoting MeXis transcription. To determine the effect of MeXis on cholesterol efflux and atherosclerosis, bone marrow from MeXis^−/−^ was transplanted into Ldlr^−/−^ mice, resulting in reduced Abca1 expression and enhanced atherosclerotic burden (66). According to the results above, MeXis was supposed to be a protective lncRNA in atherogenic progression.

### MAARS

MAARS was a macrophage-specific lncRNA identified by RNA-Seq profiling in the aortic intima of Ldlr^−/−^ mice. The level of MAARS gradually increased after the high-fat diet and descended strongly when the mice resumed a normal diet, suggesting its potential role in atherosclerosis. Knockdown studies proved MAARS to be a pro-atherogenic lncRNA due to the regression of atherosclerotic lesions. *In vitro* and *in vivo* overexpression and knockdown studies revealed that MAARS directly induced apoptosis and impaired efferocytosis of macrophages. The mechanism was that in atherosclerosis progression MAARS emerged to directly bind with HuR, an RNA-binding protein capable of stabilizing mRNA translation (67), resulting in the increase of HuR targeting genes such as p53, caspase-8,−9 related to apoptosis (68).

## Non-Coding RNAs in Hepatocytic Lipid Metabolism

### miR-33

miR-33 is one of the key regulators of lipid metabolism in both macrophages and the liver and is observed to significantly rise in human atherosclerotic plaques. By reducing ABCA1 expression in the liver or macrophages, cholesterol efflux was impaired and plasma HDL was decreased, suggesting a pro-atherogenic effect of the miRNA (75). To access its particular impact on atherosclerosis, Ldlr^−/−^ mice fed with a high-fat diet were treated with an antagonist of miR-33 for 4 weeks, resulting in reversed ABCA1 in the liver and plaque macrophages, improved plasma HDL level, and reduced lesion burden (76). In addition, miR-33 was identified to regulate mitochondrial metabolism by inhibiting mitochondrial regulatory genes related to mitochondrial respiration and ATP production, which was also contributed to cholesterol efflux in macrophages (77). Apart from the impact on lipid accumulation in macrophages, the capability of miR-33 to interfere with the balance between aerobic glycolysis and mitochondrial oxidative phosphorylation led to the suppression of M2 polarization as well as FOXP3^+^ Tregs accumulation (78). According to the results above, miR-33 would be a potential therapeutic target for atherosclerosis.

### miR-144

Two independent studies almost revealed the important role miR-144 played in cholesterol metabolism in both the liver and macrophages at the same time (79, 80). The first one found that the expression of miR-144 in the mice liver and macrophages was significantly increased by the stimulation of liver X nuclear receptor (LXR) angonists. LXRs regulated cholesterol effluxes in hepatocytes and macrophages and the plasma HDL level by regulating ABCA1 and ABCG expression. *In vivo* experiments showed that the overexpression of miR-144 could suppress ABCA1 expression and cholesterol efflux in macrophages. Similarly, overexpressing miR-144 in male C57BL/6 mice could also decrease the ABCA1 protein level in the liver and reduce plasma HDL levels, while suppressing the microRNA showed the reverse result. The later study found that the expression of miR-144 in the liver could be activated by another receptor called Farnesoid-X-Receptor (FXR) which was also highly expressed in the liver. Given the negative correlation between plasma HDL levels and atherosclerosis, miR-144 was likely to play a pro-atherogenic role, but the accurate function still reminds to be answered.

### CHROME

CHROME was a primate-specific long non-coding RNA that was found to be increased in the plasma of patients with atherosclerosis as well as in the atherosclerotic plaques. Suppression CHROME in human hepatic HepG2 cells revealed that the expression of cholesterol metabolism-related genes such as ABCA1 in hepatocytes was significantly decreased. Algorithm and *in vivo* experiments showed that CHROME could interact with miR-33, miR-27, and miR-128 and decrease their expression. These microRNAs had been proved to inhibit ABCA1 expression and promote atherogenesis (87). As a result, CHROME could promote cholesterol efflux in hepatocytes and increase plasma HDL levels. *In vivo* experiments using African green monkeys revealed that the expression of CHROME was regulated by excessive dietary cholesterol. The above results demonstrated the potential protective function of CHROME in atherosclerosis. However, the precise role it plays still needs to be evaluated (81).

### RNA Therapeutics in Atherosclerosis

Apart from small molecules and proteins, RNA drugs nowadays are attached, thereby increasing their importance as they are easy to design, synthesize, and be absorbed into the cells (88). There are various types of RNA-targeted therapeutics in atherosclerosis, such as antisense oligonucleotides (ASO), small interfering RNA (siRNA), and microRNAs (Table 2) (89). Classical ASOs are short, single-stranded DNA-like molecules that are complementary to mRNA, forming a DNA–RNA duplex that can be hydrolyzed by the enzyme Ribonuclease H (RNase H) (90). ASOs can also be anti-miRs targeting miRNAs within cells and blocking their functions, two examples of which are antagomiRs and locked nucleic acid (LNA)-based anti-miRs (91). siRNA are double-stranded RNA molecules with a length of 20–25 nucleotides that can mediate the degradation of complementary mRNA by an RNA-induced silencing complex (RISC)-dependent pathway (92). miRNA mimics are double-stranded RNAs that imitate native miRNAs to reverse the protective molecules within cells (89).

**Table 2:** RNA therapeutics in atherosclerosis.

**Drug name**	**Chemistry**	**Target**	**Clinical status**	**NCT number**
MRG-110	Antisense Oligonucleotides	miR-92a-3p	Phase I	[EudraCT]No.2017-004180-12
IONIS-ANGPTL3-L_Rx_	Antisense Oligonucleotides	hepatic *ANGPTL3 mRNA*	Phase I	NCT02709850
AKCEA-APOCIII-L_Rx_	Antisense Oligonucleotides	hepatic *APOC3* mRNA	Phase I/IIa	NCT02900027
Inclisiran	siRNA	hepatic *PCSK9* mRNA	Phase III	NCT03399370; NCT03400800

### Antisense Oligonucleotides

#### MRG-110

A previous study showed that miR-92a could directly bind to 3′UTR of KLF2 and KLF4, which could partially reverse endothelial inflammation in human arterial endothelial cells (HAECs) (93). miR-92a-3p was one of the atheromas in endothelial cells selected from large-scale miRNA profiling in human umbilical vein endothelial cells (HUVECs) stimulated by shear stress and oxLDL. miR-92a-3p was the most upregulated microRNAs subjected to low shear stress with oxLDL. Inhibition of it attenuates endothelial Inflammation *in vitro*. Consistently, blockade of miR-92a in Ldlr-deficient mice with a high-fat diet significantly reduced the atherosclerotic lesions (94).

According to the experimental results above, miR-92a-3p showed therapeutic potential. As a result, a locked nucleic acid-based anti-miR named MRG-110 targeting miR-92a-3p was developed to be utilized in a human study. A randomized, double-blinded clinical trial with a size of 49 persons at phase I (European Clinical Trials Database [EudraCT] No. 2017-004180-12) was conducted to estimate its functional efficiency to block miR-92a-3p. The volunteers were healthy men at 18–45 years of age without cigarette use for at least 3 months. The results showed that the amount of miR-92a-3p was decreased in the peripheral blood compartment with a low dose of MRG-110, revealing its high efficiency (95). However, the safety and its effects to treat atherosclerosis need to be accessed in later clinical trials.

#### IONIS-ANGPTL3-L_Rx_

ANGPTL3 is the gene encoding angiopoietin-like 3 protein reported to inhibit the activation of lipoprotein lipase and endothelial lipase. Once produced in the liver, ANGPTL3 is secreted for circulation and absorbed by other cells where it is cleaved by furin and PACE4, and its N-terminal domain is released to suppress the activity of lipoprotein lipase (96). Using the exome sequencing, two nonsense mutations in two patients with familial hypobetalipoproteinemia were identified, revealing the potential role of ANGPTL3 in lipid metabolism and atherosclerosis (97). To identify the hypothesis that ANGPTL3 deficiency protects atherogenesis, individuals with loss-of-function mutations as well as Angptl3 knockout mice were both tested for the size of their coronary atherosclerotic lesions, proving its protective role in atherosclerosis (98).

IONIS-ANGPTL3-L_Rx_ is an antisense oligonucleotide targeting hepatic ANGPTL3 mRNA to reduce ANGPTL3 translation, decrease triglycerides and LDL cholesterol in the blood, and eventually slow down atherogenesis. The phase I clinical trial (NCT02709850) to access the safety, effects, kinetics, and pharmacodynamics of IONIS-ANGPTL3-L_Rx_, as well as animal experiments, have already been completed. A total of 44 volunteers with a higher fasting LDL cholesterol or triglyceride level had received a single-dose or multiple-dose ASO drug for 6 weeks before the indexes for lipid metabolism were tested, revealing a significant reduction in pro-atherogenic lipid in the blood. The results were consistent with those of Apoc3 and Ldlr double-knockout mice which also showed a reduced atherogenesis (99).

#### AKCEA-APOCIII-L_Rx_

ApoC-III is a 99-amino acid peptide with a 20 amino acid signal peptide synthesized mostly by the liver. It plays a vital role in the metabolism of triglyceride-rich lipoproteins (TRL) since it helps the assembly of VLDL in the liver, inhibits the intake of TRL in hepatocytes as long, and suppresses the activation of LPL. A high level of plasma ApoC-III is strangely related to hypertriglyceridaemia (100). To figure out its importance on cardiovascular diseases, an exome sequencing for 18,666 genes involving 3,734 participants was launched to find out mutations deactivating ApoC-III. The individuals with the loss-of-function mutations presented lower levels of triglyceride as well as the risk of coronary heart disease (101). Therefore, ApoC-III could be a potential therapeutic target for atherosclerosis treatment.

AKCEA-APOCIII-L_Rx_ is an ASO drug with a triantennary N-acetyl galactosamine (GalNAc_3_) complex at its 5' terminal for better stability targeting hepatic APOC3 mRNA. The Phase I/IIa clinical trial (NCT02900027) aimed to evaluate its efficacy, pharmacodynamics, and side effects has been conducted. A total of 56 healthy participants with high triglyceride levels were enrolled. After subcutaneous injection of the ASO drug or placebo, every 6 weeks in single-dose cohorts or every 4 weeks in multiple-dose cohorts, the laboratory parameters referred to lipid metabolism were measured 3 months later, resulting in a significant reduction of ApoC-III and an extensive improvement in pro-atherogenic lipid (102). Therefore, further clinical trials aimed to access the effects on atherosclerotic patients are worthy of launching.

### siRNAs

#### Inclisiran

Proprotein convertase subtilisin-like type 9 (PCSK9) is a 692-amino acid protein located mostly in the liver. It is a key regulator of LDL metabolism as a serine protease that can catalyze the degradation of LDL receptors in the liver (103). Its importance in LDL metabolism was first identified in a family with autosomal dominant hypercholesterolemia, where the gain-of-function missense mutations of PCSK9 caused an increase of low-density lipoprotein cholesterol levels in the blood (104). To further determine the function of PCSK9 in LDL metabolism, the coding regions of PCSK9 in 128 participants with low plasma levels of LDL were sequenced. As expected, two nonsense mutations of PCSK9 were founded, which was consistent with the result above (105). Apart from that, variants in PCSK9 were related to increased LDL cholesterol levels and higher risk for coronary artery disease (106). So PCSK9 is supposed to be an efficient therapeutic target.

Currently, there exist two kinds of drugs targeting PCSK9 recommend by AHA guidelines for patients with high levels of LDL-C, namely the evolocumab and alirocumab (34). Both of them are monoclonal anti-PCSK9 antibodies whose benefits on lowering LDL-C and risk for cardiovascular events were identified in two large clinical trials FOURIER (107) and ODYSSEY (108). Despite its excellent efficacy, two main disadvantages block their wide usage. Firstly, it is likely to be hard to adhere to the antibody treatment since the drugs are required to be injected every 2 weeks. Besides, it is not affordable for everyone because of its high price and frequent injection (109). As a result, inclisiran with lower cost and injection frequency shpuld come out to exist.

Inclisiran is a small interfering RNA targeting PCSK9. Two clinical trials at phage III, named ORION-10 (NCT03399370) and ORION-11 (NCT03400800), were completed to evaluate the benefit of inclisiran on patients with elevated LDL-C despite statin usage. The former one was launched in the United States including 1,561 participants, while the latter one was conducted in Europe and South Africa with 1,617 volunteers involved. In both studies, 300 mg of inclisiran was given to the patients through subcutaneous injections at Day 1, Day 90, and then every 6 months. After a year and a half, the percentage change in LDL-C from baseline was tested. The results showed a reduction of 52.3% in the ORION-10 trial and 49.9% in the ORION-11 trial with a few slight side effects such as injection-site adverse events, revealing the huge therapeutic potential for patients resistant to statin therapy (110).

#### Cutting-Edge RNA Therapeutics in Atherosclerosis

Conventional RNA therapies treating atherosclerosis have their disadvantages, such as the lack of target specificity, susceptibility to degradation, and massive side effects. Nanotechnology is likely to address these problems (111). Nanomedicine applies nanotechnology for disease imaging, diagnosis, and treatment. The nanoparticles are made up of biological materials such as lipid as well as inorganic nanocrystals like iron oxide, providing a stable environment for the drugs. Equipped with antibodies or antibody fragments targeting specific ligand receptors on the cellular surfaces, the risk for interfering with normal physiological processes in irrelevant cell types declines, reducing the occurrence of adverse effects (112, 113).

Due to of the broad benefits of nanomedicine, more and more studies have focused on exploring nanoparticle strategies targeting atherosclerosis, including nanomedicines containing RNA drugs. siCAM (5) was a nanoparticle-based siRNA drug targeting endothelial cells. It contains five siRNAs separately inhibiting the expression of intercellular adhesion molecules Icam1 and Icam2, Vcam1, E- and P-selectins. *In vivo* experiments showed that monocytes recruitment was significantly decreased in the atherosclerotic lesions in the ApoE-deficient mice, revealing its therapeutic potential (114). SNALP-siApoB was a stable nucleic acid lipid particle containing siRNAs directly targeting ApoB mRNA in the livers, which were not accessible by traditional therapies. The results revealed that this nanoparticle drug could significantly reduce the ApoB expression and serum cholesterol and LDL levels for 11 days in cynomolgus monkeys (115).

## Conclusion

The above studies have demonstrated that ncRNAs play a vital role during atherogenesis. Atherosclerosis is a complicated disease where different kinds of cell types participated in its development. Among them, three kinds of cells are considered to play important roles in atherogenesis, namely endothelial cells, macrophages, and vascular smooth muscle cells. After lipid retention and oxidation under endothelium of arterials, ECs begin dysfunction, resulting in impaired permeability and overexpression of adhesion molecules. The dysfunction of ECs recruits monocytes from circulation to the lesion, where they differentiate into macrophages. The macrophages take in a mass of ox-LDL underneath the intima, resulting in impaired cholesterol metabolism within the cells, which promotes the formation of foam cells. The accumulation of foam cells forms the necrotic cores in the plaque. During the process, VSMCs continue to migrate from the media to the intima, switch their phenotypes, and produce an extracellular matrix, forming the fibrosis cap. However, their constant loss by apoptosis and necrosis finally leads to plaque rupture. ncRNAs participated in regulating all the mentioned cellular progress.

Apart from regulating the pathophysiological development of atherosclerosis, ncRNAs can also serve as therapeutic tools to treat this disease. The most successful RNA drug in atherosclerosis is Inclisiran, the siRNA drug targeting PCSK9. It has entered phase III clinical trial and showed a strong ability to reduce LDL levels. In addition to traditional RNA therapeutic strategies, advancing technologies are also used to improve the treatment effects. For example, RNA drugs can be encapsulated into nanoparticles to improve their specificity and stability, revealing the value of research to combine the RNA drugs and nanomedicines together.

## Author Contributions

YT drafted the manuscript. HL and CC supervised the manuscript. All authors contributed to the article and approved the submitted version.

## Funding

This work was supported by grants from the National Natural Science Foundation of China (nos. 81822002, 31771264, and 82170273). The funders had no role in the study design, data collection, analysis, manuscript preparation, or decision to publish.

## Conflict of Interest

The authors declare that the research was conducted in the absence of any commercial or financial relationships that could be construed as a potential conflict of interest.

## Publisher's Note

All claims expressed in this article are solely those of the authors and do not necessarily represent those of their affiliated organizations, or those of the publisher, the editors and the reviewers. Any product that may be evaluated in this article, or claim that may be made by its manufacturer, is not guaranteed or endorsed by the publisher.
